# Elucidation of the Metabolite Profile of *Yucca gigantea* and Assessment of Its Cytotoxic, Antimicrobial, and Anti-Inflammatory Activities

**DOI:** 10.3390/molecules27041329

**Published:** 2022-02-16

**Authors:** Nashwah G. M. Attallah, Suzy A. El-Sherbeni, Aya H. El-Kadem, Engy Elekhnawy, Thanaa A. El-Masry, Elshaymaa I. Elmongy, Najla Altwaijry, Walaa A. Negm

**Affiliations:** 1 Department of Pharmaceutical Science, College of Pharmacy, Princess Nourah bint Abdulrahman University, P.O. Box 84428, Riyadh 11671, Saudi Arabia; ngmohamed@pnu.edu.sa (N.G.M.A.); eielmongy@pnu.edu.sa (E.I.E.); naaltwaijry@pnu.edu.sa (N.A.); 2 Pharmacognosy Department, Faculty of Pharmacy, Tanta University, Tanta 31111, Egypt; 3 Department of Pharmacology and Toxicology, Faculty of Pharmacy, Tanta University, Tanta 31111, Egypt; aya.elkadeem@pharm.tanta.edu.eg (A.H.E.-K.); thanaa.elmasri@pharm.tanta.edu.eg (T.A.E.-M.); 4 Pharmaceutical Microbiology Department, Faculty of Pharmacy, Tanta University, Tanta 31111, Egypt

**Keywords:** carrageenan, LC-MS/MS, MIC, NO, PGE-2, TNF-α, *Yucca elephantipes*

## Abstract

The acute inflammation process is explained by numerous hypotheses, including oxidative stress, enzyme stimulation, and the generation of pro-inflammatory cytokines. The anti-inflammatory activity of *Yucca gigantea* methanol extract (YGME) against carrageenan-induced acute inflammation and possible underlying mechanisms was investigated. The phytochemical profile, cytotoxic, and antimicrobial activities were also explored. LC-MS/MS was utilized to investigate the chemical composition of YGME, and 29 compounds were tentatively identified. In addition, the isolation of luteolin-7-*O*-β-d-glucoside, apigenin-7-*O*-β-d-glucoside, and kaempferol-3-*O*-α-l-rhamnoside was performed for the first time from the studied plant. Inflammation was induced by subcutaneous injection of 100 μL of 1% carrageenan sodium. Rats were treated orally with YGME 100, 200 mg/kg, celecoxib (50 mg/kg), and saline, respectively, one hour before carrageenan injection. The average volume of paws edema and weight were measured at several time intervals. Levels of NO, GSH, TNF-α, PGE-2, serum IL-1β, IL-6 were measured. In additionally, COX-2 immunostaining and histopathological examination of paw tissue were performed. YGME displayed a potent anti-inflammatory influence by reducing paws edema, PGE-2, TNF-α, NO production, serum IL-6, IL-1β, and COX-2 immunostaining. Furthermore, it replenished the diminished paw GSH contents and improved the histopathological findings. The best cytotoxic effect of YGME was against human melanoma cell line (A365) and osteosarcoma cell line (MG-63). Moreover, the antimicrobial potential of the extract was evaluated against bacterial and fungal isolates. It showed potent activity against Gram-negative, Gram-positive, and fungal *Candida albicans* isolates. The promoting multiple effects of YGME could be beneficial in the treatment of different ailments based on its anti-inflammatory, antimicrobial, and cytotoxic effects.

## 1. Introduction

Yucca is a genus of perennial trees and shrubs that belongs to the Asparagaceae family and the Agavoideae subfamily [1]. Yucca contains approximately 50 species that can be distributed from northern Canada to the southern and southwestern United States, Mexico, and the Caribbean [2]. Furthermore, certain species, as well as various cultivars, have flooded the global horticultural market, as several Yuccas can withstand relatively severe temperate climates [2,3]. The Yucca genus has long been utilized in folk medicine. Native Americans valued the ethnobotanical significance of these native flowering plants [4,5]. *Yucca gigantea* Lem. (a synonym of *Yucca elephantipes*) has many traditional uses as a source of fibers for textiles, basketry, and cordage, as well as food [6]. In addition to the functional food potential of *Yucca* species, antioxidant, anti-cancer, antidiabetic, antibacterial, anti-arthritic, and hypocholesterolemic characteristics have also been reported [4]. El Sayed et al [7] reported that LC-ESI-MS/MS of *Y. gigantea* (synonym *Y. elephantipes*) tentatively revealed the presence of 11 compounds. Spirostanol saponins comprised the majority of the tentatively recognized compounds: spirostan-3-ol-3-*O*-[β-d-galactopyranosyl-(1→4)-β-d-glucopyranoside, gallic acid, hecogenin-rhamnoside, (3β, 5α, 25*R*)-Spirostan-3-ol-3-*O*-[β-d-glucopyranosyl-(1→2)-β-dglucopyranoside, (3β, 5β, 25*R*)-Spirostan-3-ol-3-*O*-[β-d-glucopyranosyl-(1→2)-β-dglucopyranoside], (3β, 5α, 25*S*)-Spirostan-3-ol-3-*O*-[β-d-glucopyranosyl-(1→2)-β-dglucopyranoside], (3β, 5β, 25*S*)-Spirostan-3-ol-3-*O*-[β-d-glucopyranosyl-(1→2)-β-dglucopyranoside], 25*R*-Spirostan-diol-dihexoside isomer, 25*S*-Spirostan-diol-dihexoside isomer, chlorogenic acid and cinnamic acid. In our study, we investigated the chemical composition of *Y. gigantea* in detail using the negative ion mode of LC-ESI-MS/MS, which tentatively revealed the presence of 29 different compounds. Zhang et al. [8] reported the isolation of elephanosides G and H, new spirostanol saponins, from the leaves of *Yucca elephantipes*. Two known furostanol saponins and six known flavonoid glycosides were also separated. The isolated flavonoid glycosides were kaempferol 3-{*O*-β-d-glucopyranosyl-(1→2)-*O*-[α-l-rhamnopyranosyl-(1→6)]-β-d-galactopyranoside}, 6,8-bis(β-d-glucopyranosyl)apigenin, 8-(2-*O*-α-l-rhamnopyranoyl-β-d-glucopyranosyl) apigenin, (rutin), 4′-*O*-methylquercetin 3-(6-*O*-α-l-rhamnopyranosyl-β-d-glucopyranoside), quercetin 3-{*O*-β-d-glucopyranosyl-(1→2)-*O*-[α-l-rhamnopyranosyl-(1→6)]-β-d-galactopyranoside}. In the present study, we isolated other known flavonoid glycosides.

Inflammation is a protective body response to a variety of boosts, including microbial infection, chemical irritants, and tissue injury. By being a defense mechanism, many reactions can be induced or aggravated by the vast array of events and mediators involved in the inflammatory response [9]. Numerous proinflammatory mediators, such as interleukin-6 (IL-6) and tumor necrosis factor-alpha (TNF-α), are created throughout the inflammatory process [10] as they trigger and amplify the course of inflammation [11]. In addition, various signaling molecules and enzymatic pathways participate in driving the inflammation, including cyclooxygenase 2 (COX-2), a crucial enzyme that regulates the synthesis of prostaglandins (PGs) during inflammation [12]. In addition, TNF-α and interleukin 1 beta (IL-1β) were investigated as the essential main mediators that contribute to acute and chronic inflammation [13]. Reactive oxygen species (ROS) play a critical role in cellular defense systems as they are secreted by the inflammatory cells and exacerbate the oxidative stress process [14]. ROS can also activate the intracellular signaling pathways and boost pro-inflammatory cytokine gene production [9,15].

Finding safe and efficacious drugs to limit inflammation has proven to be difficult; hence, numerous animal models have been developed for evaluating anti-inflammatory medication effects. The most extensively used model to explore the anti-inflammatory potential of several natural and synthetic compounds is the carrageenan-induced acute inflammation model [16]. Although numerous drugs are available to treat inflammatory diseases, their long-term use induces severe adverse effects. Nonsteroidal anti-inflammatory drugs are commonly used to decrease inflammation. However, their long-term usage is linked to cardiac, renal, and gastrointestinal toxicity [17]. The development of safer anti-inflammatory agents is an important subject. The sensible and effective technique for treating inflammatory illnesses is the discovery of anti-inflammatory drugs obtained from natural sources [9].

Natural products have recently become a key source of pharmaceuticals and are currently being investigated as a medical candidate for their anti-inflammatory and antimicrobial effects. They have many advantages of being safe, effective, biocompatible, and cost-efficient alternatives. Other Yucca species were medicinally known to treat osteoarthritis, inflammation, and infections [18]. In addition, they protect the skin and treat skin sores and infections [19]. Few studies have investigated the different Yucca species introduced to Egypt [7]. Therefore, the antimicrobial potential of *Y. gigantea* methanol extract of leaves (YGME) was tested against certain pathogenic microbial isolates by the agar well diffusion method. We also investigated the anti-inflammatory and cytotoxic effects of *Y. gigantea* methanol extract.

## 2. Materials and Methods

### 2.1. Plant Materials, Extraction, and Isolation of Pure Compounds

*Yucca gigantea* leaves were gathered from a nursery at Al Qanatir Al Khayriyyah, El-Qalyubia Governorate on 13th December 2020. The plant was recognized by Dr. Esraa Ammar, Plant Ecology lecturer, Botany Department, Faculty of Science, Tanta University. A voucher sample (PG-M-0069) was deposited at the Herbarium of the Pharmacognosy Department, Tanta University. The plant was dried at room temperature, then powdered. The *Y. gigantea* powder (1.2 kg) was extracted by 95% methanol (4 L × 3 times) using the cold maceration method to yield 90.3 g of YGME.

Total methanolic extract (50 g) was suspended in deionized water and applied to the Diaion HP-20 column. The column was initially eluted with deionized water, followed by 100% methanol (MeOH). MeOH fraction (21.5 g) was resuspended in aqueous MeOH (50%) and successively partitioned with dichloromethane (CH_2_Cl_2_), ethyl acetate (EtOAc), and *n*-butanol (*n*-BuOH). Based on thin-layer chromatography (Merck, Darmstad, Germany), EtOAc fraction (9.1 g) was subjected to further investigation and chromatographed over vacuum liquid chromatography (silica gel 50 g, ϕ 4 cm × 11 cm) eluted with CH_2_Cl_2_ before adding MeOH in 1% increments. After TLC observation using Camag UV lamp at 254 and 366 nm, fractions were divided into four groups, from YE-1 to YE-4. Fraction YE-2 (518 mg) was rechromatographed over (silica gel 20 g, ϕ 1 cm × 25 cm) column, then purified using Sephadex LH-20 (Merck, Darmstad, Germany) with methanol to afford compounds I and II (11 and 9 mg, respectively). Isocratic CC on silica gel with (95:5) CH_2_Cl_2_: MeOH was performed on fraction YE-3 (260 mg) to give compound III (13 mg). A High-Performance Digital FT-Avance III NMR spectrometer (Bruker, Karlsruhe, Germany) was used to record NMR spectra. The ^1^H and ^13^C-NMR samples were examined at 400 MHz and 100 MHz, respectively. DMSO-*d*_6_ was used to dissolve the samples.

### 2.2. Animals

The animal house at Cairo University’s College of Veterinary Medicine provided a total of 40 male Wistar albino rats with a weight of 170–210 g. All rats were housed in normal rat cages with ad libitum access to standard pellet food and filtered water under controlled temperature (25 ± 2 °C) and illumination (12 h-light/dark cycle). Before being used in experiments, all rats were given a one-week acclimatization period. The Research Ethical Committee authorized all methods and experimental protocols in conformity with the rules for the care and use of laboratory animals (Faculty of Pharmacy, Tanta University, Egypt).

### 2.3. Materials, Drugs and Chemicals

Celecoxib was purchased from Amoun Pharma (Cairo, Egypt). All other chemicals and solvents used were bought from Merck (Kenilworth, NJ, USA) and were of a high analytical grade.

### 2.4. LC-MS/MS for Metabolite Analysis

#### 2.4.1. Sample Preparations

The sample was prepared by macerating *Yucca gigantea* powder at room temperature in mild petroleum ether. The powder was extracted with MeOH after exhaustion, then the extract was evaporated under vacuum at 45 °C. A weighed amount of the dry residue (50 mg) was then added to a 1 mL solution of deionized water:methanol:acetonitrile (50:25:25). This mixture was vortexed for 2 min, ultra-sonicated for 10 min, then centrifuged for another 10 min at 1000 rpm. The sample solution was diluted with the reconstitution solvent and 10 L was injected at a concentration of 1 g/L [20].

#### 2.4.2. Acquisition Method and Analytical Parameters

Analysis was carried out in the Proteomics and Metabolomics Unit, Children’s Cancer Hospital (57357), Basic Research Department, Cairo, Egypt. Adopting the methods previously reported by Negm et al. [21]. In-line filter disks (0.5 µm × 3.0 mm, Phenomenex^®^, Torrance, CA, USA) and X select HSS T3 (2.5 µm, 2.1 mm × 150 mm, Waters^®^, 40 °C, Milford, MA, USA) were used as a pre-column and analytical column, respectively. The mobile phases consisted of buffer A (5 mM ammonium formate buffer pH 3 containing 1% methanol), buffer B (5 mM ammonium formate buffer pH 8 containing 1% methanol), and buffer C (100 % acetonitrile). The flow rate was adjusted at 0.3 mL/min. The liquid chromatography (ExionLC -High flow LC, Sciex^®^) was programmed to use a mobile phase composition of buffer A and C in positive mode, and buffer B and C in negative mode. The mobile phase composition started with 90 (A or B): 10 (C) for the first 20 min, which was inversed from 21–25 min, and finally returning to the starting values for the last 3 min until the end of the protocol at 28 min. In addition, the instrument (ExionLC -High flow LC-, Sciex®, Framingham, MA, USA) was coupled with Triple TOF 5600+ (Sciex^®^) for IDA acquisition and Analyst TF 1.7.1 (Sciex^®^) for LC-Triple TOF control.

#### 2.4.3. Data Processing

MasterView was used for feature (peaks) extraction from Total ion chromatogram (TIC) based on a signal-to-noise of greater than 5 (non-targeted analysis) and sample-to-blank intensities of greater than 3. In addition, Reifycs Abf (Analysis Base File) Converter (Reifycs^®^) was applied for Wiff file conversion and MS-DIAL 4.6 (RIKEN^®^) for data analysis. The ReSpect Database used possessed 1573 and 2737 records for negative mode, respectively. Metabolite’s annotation was conducted with the ReSpect Database and fragmentation pattern and retention times mentioned in previous reports for metabolites isolated from the investigated plant or others [7,22].

### 2.5. Cell Lines

Nawah Scientific Inc., (Mokatam, Cairo, Egypt) provided A375 (human melanoma), Hep-2 (head and neck cancer), A431 (human epidermoid skin carcinoma), MG-63 (osteosarcoma), and HSF (human skin fibroblast) cell lines. The cells were cultured in a humidified, 5% (*v*/*v*) CO_2_ atmosphere at 37 °C.

### 2.6. In Vitro Cytotoxicity

Sulforhodamine B (SRB) assay was carried out to determine the in vitro cytotoxicity of YGME. In 96-well plates, 100 μL cell suspension aliquots (5 × 10^3^ cells) were cultured in complete media for 24 h. Other aliquots of 100 μL media, containing various concentrations (0.03, 0.3, 3, 30, 300 µg/mL of YGME, were added to the cells. After 72 h of drug treatment, the cells were fixed by adding 150 μL of 10% trichloroacetic acid to the media and incubating for one hour at 4 °C. The trichloroacetic acid solution was taken out and distilled water was used to wash the cells five times. Then, 70 μL SRB solution (0.4 % *w*/*v*) was added in aliquots and incubated for 10 min in the dark. Using acetic acid (1%), plates were washed three times and 150 μL of TRIS (10 mM) was added to dissolve the protein-bound SRB stain. At 540 nm, the absorbance was measured using a microplate reader (BMGLABTECH^®^ FLUO-star Omega, Ortenberg, Germany) [23].

### 2.7. In Vitro Antimicrobial Activity

#### 2.7.1. Agar Well Diffusion Method

Using the agar well diffusion method, the antimicrobial activity of YGME extract was evaluated [24,25]. The tested bacterial and fungal species were *Escherichia coli* (ATCC 25922), *Klebsiella pneumoniae* (ATCC 13883), *Pseudomonas aeruginosa* (ATCC 27853), *Proteus mirabilis* (ATCC 35659), *Salmonella typhimurium* (ATCC 14028) as Gram-negative bacteria. In addition, *Staphylococcus aureus* (ATCC 25923) and *Staphylococcus epidermidis* (ATCC 6538) as Gram-positive bacteria. *Candida albicans* (ATCC 90028) was used as an example for fungi. On Muller–Hinton agar (MHA) plates, the bacterial isolates were streaked using sterile cotton swabs. A sterile cork borer was used to make 6 mm diameter wells in the agar plates, and 100 µL of 2 mg/mL (w/v) extract was applied to the wells. The agar plates were then incubated at 37 °C for 24 h using 0.1% chlorhexidine as a positive control and DMSO as a negative control, with a concentration of 10%. The appearance of clear zones indicated inhibition of bacterial growth and the zone diameters were measured in millimeters. Both 2% glucose and 5 µg/mL methylene blue were added to MHA to test the antifungal activity [26].

#### 2.7.2. Determination of MIC Values

The broth microdilution method was employed to assess the MIC values [27]. One hundred microliters were taken from the stock solution of the extract (1000 µg/mL) and two-fold diluted with 100 µL of the test organism in Muller–Hinton broth (MHB). Each microtitration pate contained a negative growth control well (MHB only) and a positive control well (bacteria only). The microtitration plates were aerobically incubated for 24 h at 37 °C. The MIC of YGME is the lowest concentration at which observable bacteria growth is completely inhibited.

### 2.8. In Vivo Anti-Inflammatory Activity

#### 2.8.1. Induction and Assessment of Carrageenan-Induced Paw Edema

In our study, rats were divided into five groups (8 rats/group). Group I: normal control group was administered 0.9% saline (10 mL/kg p.o). Groups II (positive control) and III (standard treatment) were orally treated with 0.9% saline and celecoxib (50 mg/kg) [11], respectively. Groups IV and V were orally treated with YGME (100 and 200 mg/kg, respectively). Doses of YGME were chosen based on the results of our pilot study and doses used in other Yucca species [28]. After 1 h, acute inflammation was triggered by subcutaneous injection of 100 μL of 1% carrageenan sodium in 0.9% saline into the plantar side of the right hind paw of rats in all groups except group I [29]. The thickness of the paws was measured at 0 h, and then 1, 2, 3 and 4 h later, to measure the increase in paw thickness [30]. At the end of the experiment, the weight of both the left and right paws was determined. The difference between the right and left paw weights was used to calculate the edema weight [31].

#### 2.8.2. Sample Collection

Rats were anesthetized with diethyl ether, 4 h after receiving carrageenan, and blood was obtained through heart puncture into a syringe and centrifuged for 10 min at 3000 rpm. The serum was properly removed and stored at −20 °C to measure IL-6 and IL-1β. The rats were subsequently sacrificed by cervical dislocation under light ether anesthesia, and both paws were removed and weighed to determine the weight of the edema. The paw tissue was then separated, and one part was snap-frozen in liquid nitrogen to measure reduced glutathione (GSH), nitric oxide (NO), myeloperoxidase (MPO), TNF-α, and prostaglandin E2 (PGE2). The other part was used for immunohistochemical staining and histopathological examination.

#### 2.8.3. Determination of Paw GSH Concentration

Reduced glutathione was spectrophotometrically measured in paws using the previously published method [32]. The absorbance of the yellow color, measured at 412 nm, is proportional to GSH concentration.

#### 2.8.4. Determination of Paw NO Content

Stable metabolites nitrite and nitrate were measured in paw tissue homogenate to assess the NO level, as previously described [33], using the Griess reagent, which caliometrically detects these anions. At 540 nm, the absorbance was measured and, to determine NO concentration in each sample, a sodium nitrite standard curve was established.

#### 2.8.5. Determination of Paw MPO Activity

The activity of MPO was measured using the previously reported method [34]. The kinetic measurement of yellowish-orange compounds for 5 min at 460 nm was used to develop this approach using a UV-Vis spectrophotometer (Shimadzu, Kyoto, Japan).

#### 2.8.6. Determination of the Levels of Inflammatory Markers

According to the manufacturer’s protocol, the levels of the inflammatory mediators TNF-α and PGE2 in rat paw tissues were measured using commercial ELISA kits (Sun Red biotechnology Co., Ltd., Shanghai, China). In addition, the levels of IL-1β and IL-6 in the serum samples were measured using ELISA kits (Abcam corporation, Waltham, MA, USA).

#### 2.8.7. Histopathologic Examination of the Paw Tissue

Paw tissues were fixed in formalin solution and embedded in paraffin wax, serially sectioned (5 μm), and stained with hematoxylin and eosin (H&E) to assess histopathologic changes. The stained sections were examined using a light microscope.

#### 2.8.8. Immunohistochemical Determination of COX-2 Expression

The expression of COX-2 was detected by immunostaining tissue sections prepared from formalin-fixed, paraffin-embedded hind paws using a kit obtained from ABclonal Technology (Woburn, MA, USA). An immunoperoxidase (PAP, peroxidase/anti-peroxidase) technique was adopted. In this way, the cytoplasm of each COX-2 (+) cell was stained brown. The results were scored according to the percentage of positive staining for COX-2 as follows: score 0, no positive staining; mild, score 1+, from 1–10%; moderate, score 2++, from 11–50%; strong, score 3+++, more than 50% positive cells. In this study, scores of +, ++, and +++ were considered to be positive immunostaining, and a score of 0 was regarded as negative immunostaining [13].

### 2.9. Statistical Analysis

The measurements in the current study were perfumed three times and the obtained results were recorded in the form of mean ± standard deviation (SD). All standard curves were subjected to regression analysis and correlation coefficients were calculated. One-way analysis of variance (ANOVA) was used to compare distinct groups, followed by a Tukey–Kramer posthoc test. The significance level was established at (*p* < 0.05). Prism version 9 (GraphPad Software Inc., San Diego, CA, USA) was used to conduct the statistical analysis.

## 3. Results

### 3.1. LC-ESI-MS/MS Analysis of YGME

In negative mode, YGME components were tentatively identified using the liquid chromatography with mass spectrometry (LC-MS/MS) technique. Table 1 shows the presence of 29 compounds from different phytochemical subclasses and the total ion chromatogram (TIC) of YGME (negative mode) is represented in the Supplementary Materials, Figure S1. The MS/MS spectrums of the major identified compounds are displayed in Figure S2.

#### 3.1.1. Characterization of Flavonols and Flavonols Glycosides

**Figure 1:**
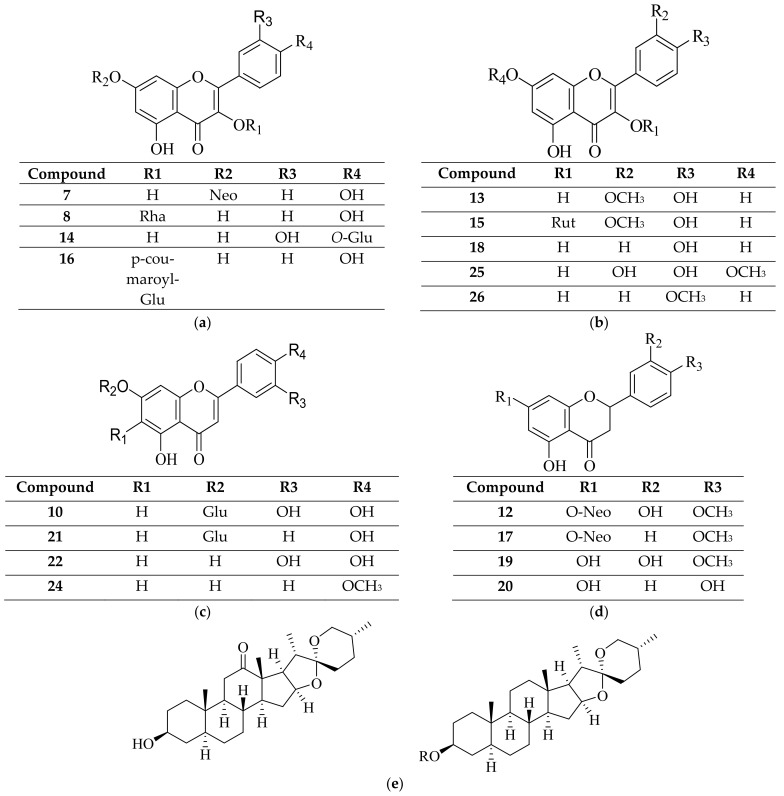

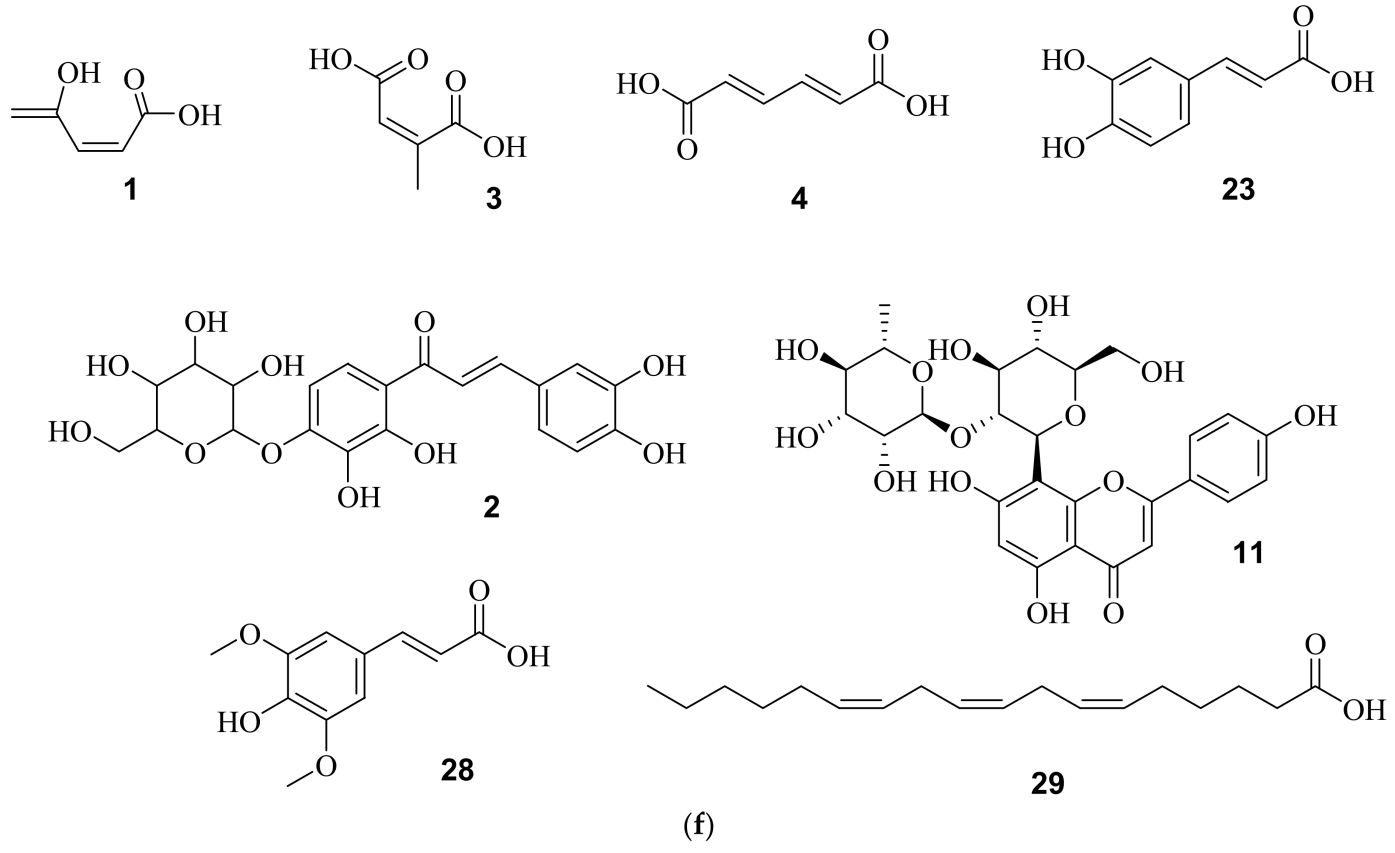
(**a**) Structures of flavonols and flavonol glycosides identified in YGME (Neo = neohesperidoside, Rha = rhamnose, Glu = glucose); (**b**) Structures of hydroxylated and/or methoxylated flavonols and flavonol glycosides identified in YGME (Rut = Rutinoside); (**c**) Structures of flavones and flavone glycosides identified in YGME; (**d**) Structures of flavanones and flavanone glycosides identified in YGME.; (**e**) Structures of saponin and saponin glycosides identified in YGME (**5** R = Glu-Gal, **9** R = Glu-Glu, **27** R = OH).; (**f**) Structures of organic, phenolic acids, and other compounds identified in YGME.

Most of the identified flavonols glycosides were found to be kaempferol derivatives. Compounds 7, 8 and 16 produced pseudo-molecular ion peaks [M − H]^−^ at *m*/*z* 593.152, 431.192, 593.519, respectively, which were ascribed to kaempferol-7-*O*-neohesperidoside, kaempferol-3-*O*-α-l-rhamnoside, kaempferol-3-*O*-(6-p-coumaroyl)-glucoside, respectively. The [M − H]^−^ of the aglycone ion (kaempferol) was recognized at *m*/*z* 285. Additionally, quercetin glycosides were identified in YGME. Quercetin-4′-*O*-glucoside showed a pseudomolecular ion peak [M − H]^−^ at *m*/*z* 463.085. The ion fragment of aglycone moiety (quercetin) was detected at *m*/*z* 301.041 [M − H-162]^−^ due to the neutral loss of glucose moiety. The chemical structures of these compounds are displayed in Figure 1a.

#### 3.1.2. Characterization of Hydroxylated and/or Methoxylated Flavonols and Flavonols Glycosides

Compounds 13, 15, 18, 25, and 26 were tentatively identified as isorhamnetin, isorhamnetin-3-*O*-rutinoside, 4′,5,7-trihydroxyflavonol, 3,3′,4′,5-tetrahydroxy-7-methoxy flavone, and 3,5,7-trihydroxy-4′-methoxyflavone, respectively, which showed deprotonated molecular ion peaks [M − H]^−^ at *m*/*z* 315.135, 623.197, 285.077, 315.092 and 299.093, respectively. The MS/MS fragment ion at *m*/*z* 315.049 [M − H-308]^−^ of isorhamnetin-3-*O*-rutinoside was due to the neutral loss of sugar moiety (rhamno-glucoside) (Figure 1b).

#### 3.1.3. Characterization of Flavones and Flavones Glycosides

The in-house database identified compounds 10, 21, 22 and 24 as Luteolin-7-*O*-β-d-glucoside, apigenin-7-*O*-β-d-glucoside, luteolin, and acacetin, respectively, based on pseudo-molecular ion peaks [M − H]^−^ at *m*/*z* 447.09, 431.17, 285.039, and 283.060, respectively (Figure 1c).

#### 3.1.4. Characterization of Flavanones and Flavanone Glycosides

The deprotonated molecular ion peaks [M − H]^−^ of compounds 12, 17, 19 and 20 were exhibited at *m*/*z* 609.147, 593.265, 301.069, and 271.060, respectively. The in-house database deduced that they were hesperetin-7-*O*-neohesperidoside, poncirin, hesperetin, and naringenin, respectively. The MS/MS fragment ions of the aglycone of hesperetin-7-*O*-neohesperidoside and poncirin were detected at *m*/*z* 301.023 and 285.038, respectively. The neutral loss of sugar moiety of neohesperidoside produced the fragment ion of the aglycone (Figure 1d).

#### 3.1.5. Characterization of Saponins

The four compounds 5, 6, 9 and 27, which are saponin derivatives, were tentatively identified. Compound 27 produced a deprotonated molecular ion peak [M − H + HCOOH]^−^ at *m*/*z* 461.26, which corresponded to the formic adduct of spirostan-3-ol, the main saponin aglycone in Yucca species [35]. Pseudo-molecular ion peaks [M − H]^−^ at m/z 739.79, attributed to spirostanol-3-ol-dihexose, were found in compounds 5 and 9. Due to the order of elution, compound 9 was thought to be the di-glucoside, while compound 5 is the glucoside-galactoside isomer [7]. Compound 6 was identified as hecogenin, an aglycone found in numerous Yucca species; the molecule produced a deprotonated molecular ion [M − H]^−^ at *m*/*z* 429.171 (Figure 1e).

#### 3.1.6. Characterization of Organic and Phenolic Acids

The in-house database identified compounds 1, 3, 4 and 23 as maleic acid, citraconic acid, muconic acid, and cafeic acid, respectively, based on the deprotonated molecular ion peaks [M − H]^−^ at 115.001, 129.019, 141.020, and 179.033, respectively (Figure 1f).

#### 3.1.7. Characterization of Other Compounds

Compound 2, a chalconoid derivative, at *m*/*z* 449.085, displays a pseudo-molecular ion peak [M-H]^−^, which was identified to be marein or okanin-4′-*O*-glucoside. Compounds 28 and 29 are 3-(4-hydroxy-3,5-dimethoxy phenyl)-2-propenoic acid and gamma-linolenic acid, respectively, with pseudo-molecular ion peaks [M − H]^−^ at *m*/*z* 223.171 and 277.197, respectively. Compound 11 is a flavonoid C-glycoside with a pseudo-molecular ion peak [M − H]^−^ at *m*/*z* 577.156 attributed to vitexin-2″-*O*-rhamnoside (Figure 1f).

### 3.2. Structure Elucidation of Compounds Isolated from YGME

**Figure 2:**
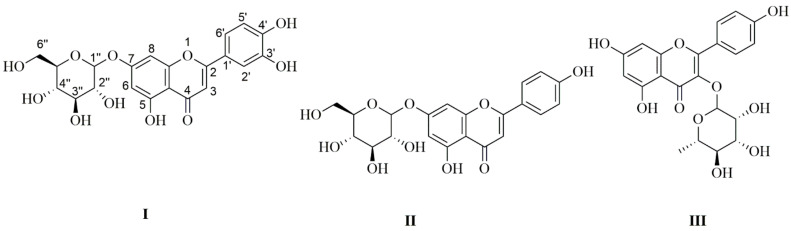
Chemical structures of the compounds isolated from YGME. Compound **I**: Luteolin-7-*O-*glucoside, **II**: Apigenin-7-*O*-β-d-glucoside, and **III**: Kaempferol-3-*O*-α-l-rhamnoside.

^1^H-NMR (DMSO-*d*_6_, 400 MHz) and ^13^C-NMR (DMSO-*d*_6_, 100 MHz) for isolated compounds were displayed in Table S1. ESI-MS *m*/*z* [M − H]^−^ were 447.09, 431.17, and 431.192 for compounds I, II, and III, respectively (Figure 2).

The ^1^H-NMR spectrum of compound I exhibited two meta–coupled doublets (*J* = 2.4 Hz) at δ_H_ 6.78 and 6.44, each integrating with one proton, and were assigned to H-8 and H-6, respectively, of ring A of 5,7-dihydroxyflavonoids. The presence of an ABX system of C6′, C5′ and C2′ at δ_H_ 7.42 of (dd, *J* = 8.0, 2.4 Hz), 7.45 (d, *J* = 2.4 Hz) and 6.91 (d, *J* = 8.0 Hz), respectively, is known for the 1,2,4-trisubstituted phenyl unit [36]. A singlet resonating at δ_H_ 6.77, integrating with one proto, was characteristic for the C-3 proton of flavonoids [37]. The luteolin skeleton was cleared with these spectral data. In addition, the anomeric proton and carbon were located at δ_H_ 4.69 and δ_C_ 99.9, respectively. the ^13^C-NMR chemical shifts in the sugar carbons were shown at δ_C_ (99.9, 73.2, 77.2, 69.6, 76.5, 60.7), revealing the presence of β-*O*-glucoside unit in luteolin-7-*O*-glucoside. The ^13^C-NMR data showed the presence of a ketone carbonyl δ_C_ 181.9, two olefinic carbons (δ_C_ 164.6 and 103.3), and four hydroxyl carbons of C5, C7, C3′, and C4′ (δ_C_ 161.2, 163.0, 145.9 and 150.9). The ESI-MS *m*/*z* [M − H]^−^ was 447.09. The previous information confirmed the structure of luteolin-7-*O-*glucoside, as reported in the literature [38].

^1^H-NMR and ^13^C-NMR spectra confirmed the presence of apigenin and glucose moieties in the structure of compound II. The apigenin skeleton was deduced from two doublets δ_H_ at 6.45 (d, *J* = 2 Hz, H-6) and δ_H_ at 6.93 (d, *J* = 2 Hz, H-8) on the A-ring; A2B2-type aromatic δ_H_ at 7.95 (d, *J* = 8 Hz, H-2′, H-6′) and δ_H_ at 6.94 (d, *J* = 8 Hz, H-3′, H-5′); together with an olefinic δ_H_ at 6.83 (s, H-3) on a flavone C-ring. Besides this, glycosidic δ_H_ at 5.06 (d, *J* = 7.2 Hz, H-1″) and δ_C_ at 99.9 (C-1″) were evident in the ^1^H and ^13^C-NMR spectra. The multiplet δ_H_ at 3.27–3.47 (5H, m, H-3″–H-6″) could be assigned to the coupling between protons and methylene protons of the glucosyl ring. Proton δ_H_ at 3.71 (m, H-2″). The ^13^C-NMR displayed 21 carbon atoms for this compound. A glucose moiety was assigned at δ_C_ 99.9, 73.2, 76.5, 69.6, 77.3, and 60.7 for C-1″–C-6″, respectively. The flavone spectrum also displayed signals at δ_C_ (161.5, 99.6, 164.3 and 94.9) for C5–C8 of A-ring, respectively. δ_C_ (163.0, 103.2, 182.1, 161.5 and 105.4) for the C2–C4, C9, C10 of C-ring, respectively. Carbons C1′–C6′ of B-ring were displayed at δ_C_ (121.1, 128.7, 116.1 and 161.2). The ESI-MS *m*/*z* [M − H]^−^ was 431.17. The spectral data fit with the data that were previously reported in the literature. Therefore, the structure of the isolated compound was elucidated as apigenin- 7-*O*-β-d-glycoside [39].

^1^H-NMR spectrum of compound III showed four aromatic hydrogen signals. Two protons at δ_H_ 7.77 (2H, dd, *J* = 8.0, 1.6 Hz, H-2′ and H-6′) and two protons at δ_H_ 6.92 (2H, dd, *J* = 8.0, 1.6 Hz, H-3′and H-5′) showed a symmetric pattern with substitution at 1 and 4 positions of B-ring. Protons at H-2′ and H-3′ were ortho-coupled as well as at H-5′ and H-6′ (*J* = 8 Hz), while the proton at H-2′ meta-coupled with H-6′, and H-3′ with H-5′ (*J* = 1.6 Hz) of flavone skeleton. The other two aromatic proton signals of H-8 and H-6 were displayed at δ_H_ 6.43 and 6.21, respectively. The anomeric proton was located at δ_H_ 5.29 and δ_C_ 102.2. The ^13^C-NMR spectrum indicated 19 carbon signals. The spectrum displayed trihydroxy substitutions at δ_C_ of C-5 (161.7), C-7 (164.1), and C-4′ (160.4) in the flavone skeleton. The deoxy carbon C-6″ was resonating at δ_C_ 17.9, in addition to the other carbons C-1″–C-5″, which were resonating at 102.2, 70.6, 70.8, 71.5, and 71.1, respectively. This indicated the occurrence of the rhamnosyl unit in this compound. The ESI-MS *m*/*z* [M − H]^−^ was 431.192. Following the literature data, this compound was identified as kaempferol-3-*O*-α-l-rhamnoside (afzelin) [40]. Thin-layer chromatography was used to compare the R_f_ values of the three isolated flavonoid glycosides to authentic samples. This led to another confirmation of the elucidated structure.

### 3.3. In Vitro Cytotoxic Activity of YGME

**Figure 3:**
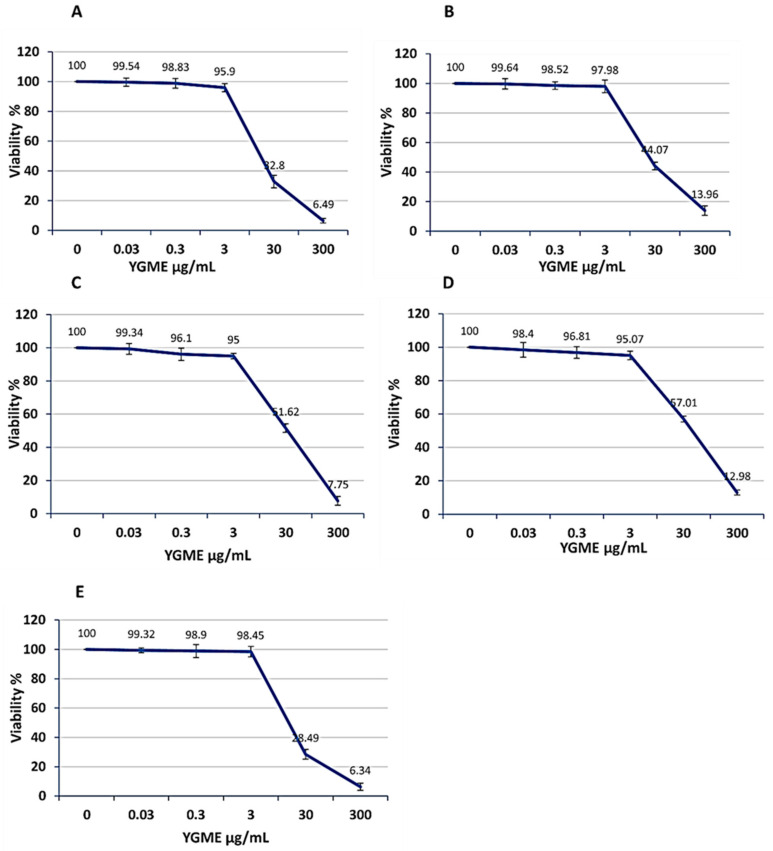
SRB cell viability assay for determination of the cytotoxicity of YGME, at different concentrations, on (**A**) A375; (**B**) Hep-2; (**C**) HSF; (**D**) A431 and (**E**) MG-63 cell lines. Values are presented as mean ± SD of 3 independent experiments.

**Figure 4:**
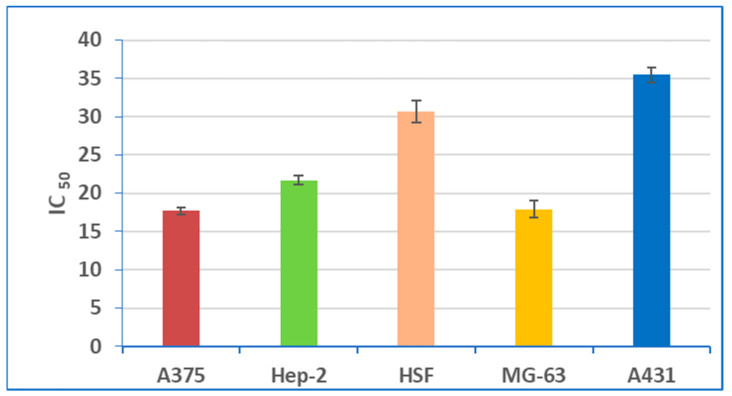
The values of IC_50_ of YGME against different investigated cell lines.

Using SRB cell viability assay, IC_50_ (half-maximal concentration) of YGME was determined. The values of IC_50_ were 17.7 ± 0.43, 21.7 ± 0.556, 17.9 ± 1.059, 35.4 ± 0.984, and 30.7 ± 1.425 µg/mL against A375, Hep-2, MG-63, A431, and HSF cell lines, respectively. The best cytotoxic activity was recorded against A-375 and MG-63 cell lines. The results of the SRB cell viability assay are displayed in Figure 3 and the values of IC_50_ of YGME against the different investigated cell lines are presented in Figure 4.

### 3.4. In Vitro Antimicrobial Activity of YGME

In the current study, we investigated the antibacterial activity of *Y. gigantea* methanol extract of leaves (YGME) using the agar well diffusion method, as shown in Table 2, against Gram-negative standard isolates, including *Escherichia coli* (ATCC 25922), *Klebsiella pneumoniae* (ATCC 13883), *Pseudomonas aeruginosa* (ATCC 27853), *Proteus mirabilis* (ATCC 35659), *Salmonella typhimurium* (ATCC 14028), and Gram-positive standard isolates including *Staphylococcus aureus* (ATCC 25923), *Staphylococcus epidermidis* (ATCC 6538). In addition, an antifungal activity against *Candida albicans* (ATCC 90028) was found. Moreover, the minimum inhibitory concentrations of YGME were determined against the aforementioned isolates. The study performed by Mahjbeen et al. [41] determined an antibacterial activity against Gram-positive bacteria (*S. aureus*, *B. subtilis*) as well as Gram-negative bacteria (*E. coli*, *P. aeruginosa*) using the disc-diffusion method. In our study, we tested the antibacterial activity against a larger number of Gram-negative isolates and tested the antifungal activity of YGME in addition to measuring the MIC values.

### 3.5. In Vivo Anti-Inflammatory Activity

#### 3.5.1. Effect of YGME on the Average Edema Volume

As shown in Table 3, the carrageenan group induced severe paw edema, which attained a peak level of 4 h after carrageenan administration. The edema volume (mm) was measured after different time intervals (1, 2, 3 and 4 h) in all studied groups. After 1 h, the average change in the edema volume showed a higher level in group II, while the edema volume in groups III, IV, and V had a lower paw volume. Similar results were observed after 2, 3 and 4 h of the experiment, with the highest paw volume was observed in group II, and a significant reduction in the edema volume seen in group V. These findings demonstrated that YGME produced a pronounced decline in the edema volume compared to the other treatments at *p* < 0.05.

#### 3.5.2. Effect of YGME on the Average Paw Edema Weight

In comparison with group I, group II exhibited a significant increase in the paw edema weight, with a percentage of 1133.3%. Treatment with celecoxib (group III) presented a significant reduction in the edema weight compared to the carrageenan group (group II), with a percentage of 59.45%. Treatment with YGME 100 (group IV) and 200 (group V) significantly decreased the edema weight, with percentages of 54.05, and 81.08%, respectively, compared to group II. The decrease in the edema weight in group V was superior, as shown in Table 4 (*p* ˂ 0.05).

#### 3.5.3. Effect of YGME on MPO Activity

In comparison with group I, group II presented a marked increase in MPO activity, with a percentage of 388.3%. Group III exhibited a pronounced decrease in MPO activity compared to group II, with a percentage of 66.46%, while groups IV and V showed a significantly suppressed MPO activity (55.31 and 75.91%, respectively) when compared to group II, with the results being superior in group V, as shown in Table 4 (*p* ˂ 0.05).

#### 3.5.4. Effect of YGME on the Oxidative Stress Markers

By comparison to the group I, group II revealed massive oxidative damage, as evident by a considerable reduction in GSH content in the paw tissue (30.12%). Markedly increased GSH levels (7.6%) were found in group III compared to group II. In addition, groups IV and V showed significant restoration of the GSH levels (20.51, and 77.91%, respectively) compared to group II, with a more prominent effect in group V, as shown in Table 4 (*p* ˂ 0.05).

Group II exhibited a significant elevation in the paw NO content (90.56%) in comparison to group I. Group III revealed a significant reduction in the NO levels (46.53%) compared to the group II. In addition, treatment with YGME 100 and 200 (groups IV and V) induced a significant suppression of NO levels (32.67 and 48.01%, respectively) compared to group II, with a more significant result in group V, as shown in Table 4 (*p* ˂ 0.05).

#### 3.5.5. Effect of YGME on the Levels of the Inflammation Markers

**Figure 5:**
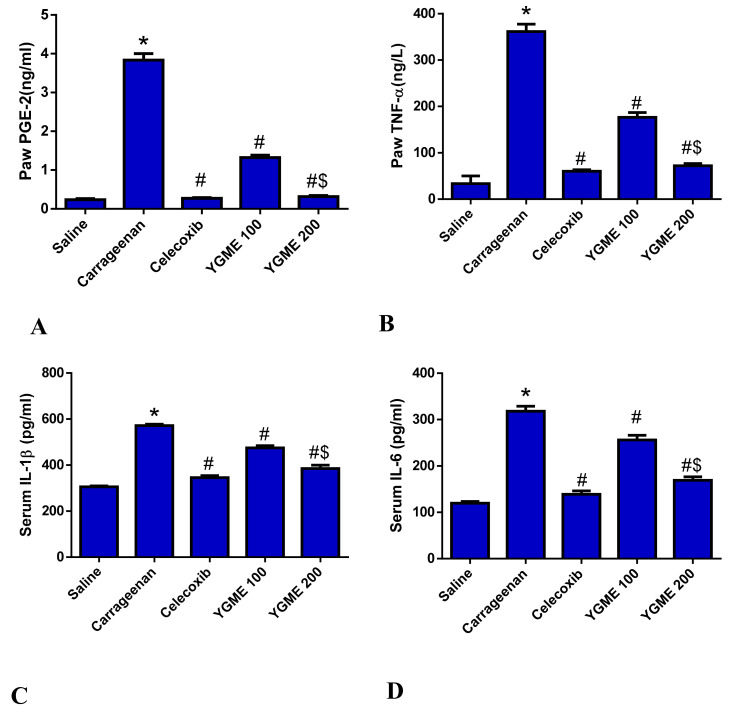
Drug treatment effects on (**A**) paw PGE-2 level, (**B**) paw TNF-α level, (**C**) serum IL-1β level, (**D**) serum IL-6 level. Data expressed as mean ± SD (*n* = 8/group). Significant difference vs. * respective control saline, # respective carrageenan group, $ respective YGME 100 group, each at *p* ˂ 0.05.

Group II showed a marked inflammation, demonstrated by the remarkable increase in TNF-α content (799%) in comparison to group I. Celecoxib treatment (group III) significantly reduced TNF-α (83.34%) compared to the group II, while treatment with YGME 100 and 200 (groups IV and V) induced a considerable reduction in TNF-α levels (51.13 and 80%, respectively) compared to group II, with a more pronounced effect in group V, as shown in Figure 5 (*p* ˂ 0.05).

Group II also displayed a marked increase in IL-1β and IL-6 serum levels (86.98 and 165.25%, respectively) in comparison to group I. Celecoxib treatment (group III) strongly reduced IL-1β and IL-6 levels (39.52 and 56.39%, respectively) compared to group II. In addition, treatment with YGME 100 and 200 (groups IV and V) induced a significant suppression of IL-1β levels (16.94, 19.54% and 32.65, 46.9%, respectively) in comparison with group II, with a more pronounced effect in group V, as shown in Figure 5 (*p* ˂ 0.05).

#### 3.5.6. Effect of YGME on Paw PGE-2 Levels

The current study showed a marked-up regulation in paw PGE-2 levels (1522%) in group II compared to group I. Celecoxib treatment (group III) significantly reduced PGE-2 levels (92.89%), compared to group II. In addition, treatment with YGME 100 and 200 (groups IV and V) induced a marked decrease in PGE-2 levels (65.43 and 91.74%, respectively) in comparison to group II. The results were more significant in group V, as shown in Figure 5 (*p* ˂ 0.05).

#### 3.5.7. Effect of YGME on the Histopathology of Paw Tissues

**Figure 6:**
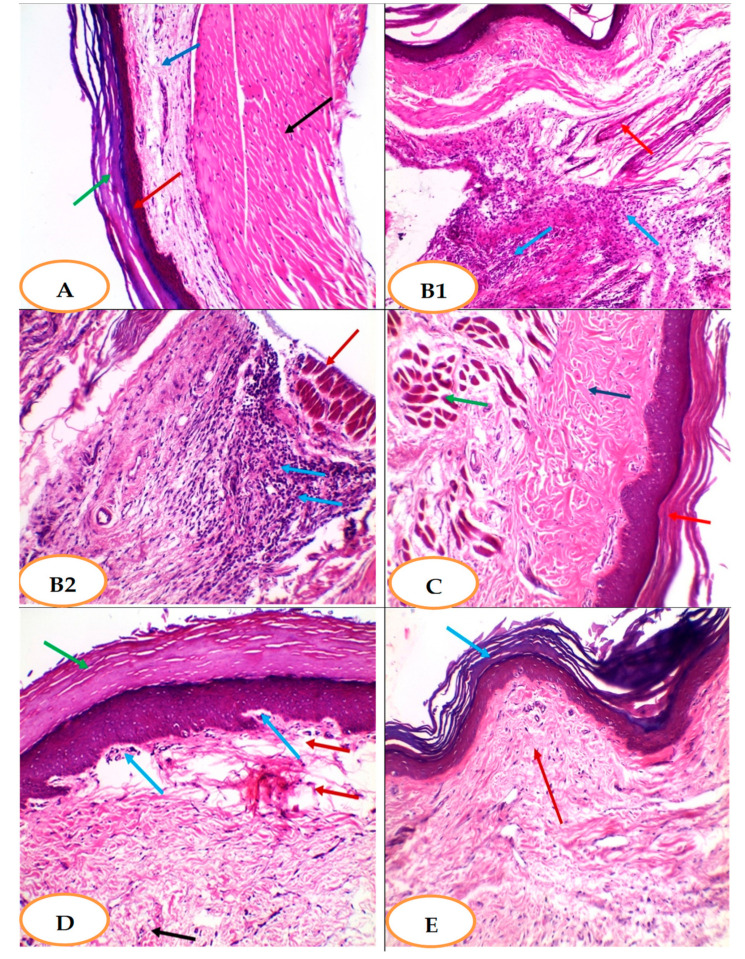
Effects of different treatments on histopathological examination of paw tissues. (**A**): Section in paw skin of the normal control group showed normal skin consisting of epidermis of average thickness (red arrow) lined with thick keratin (green arrow) and underlying normal dermis (blue arrow) and normal muscles (black arrow) [H&E × 100]. (**B1**): section in paw tissue of Carrageenan group [positive control group] showed deep, dense dermal infiltration with chronic inflammatory cells (blue arrows) with congested vessel (red arrow) [H&E × 100]. (**B2**): section in paw tissue of Carrageenan group [positive control group] showed severe interstitial inflammatory reaction (blue arrows) between muscle bundles (red arrow) [H&E × 200]. (**C**): section in paw tissue of celecoxib-50-treated group showed no inflammation. Muscles are normal (green arrow); however, the epidermis was thickened and covered with excessive keratosis (red arrow) with underlying excessive collagenosis (blue arrow) [H&E × 200]. (**D**): Section in paw tissue of YGME 100 showed superficial dermal mild chronic inflammation (blue arrows) with mild edema (red arrows) and mild vascular congestion (black arrow); the epidermis was covered with excessive keratosis (green arrow) [H&E ×200]. (**E**): Section in paw tissue of YGME 200 Group showed normal skin without inflammation or collagenosis, with normal epidermis lined with thick keratin (blue arrow) and dermis (red arrow) [H&E × 200].

The section in the normal control group (group I) paw showed normal skin consisting of epidermis of average thickness lined with thick keratin and underlying normal dermis and normal muscles **(**Figure 6A). The section in the carrageenan group (group II) showed deep dense dermal infiltration, with chronic inflammatory cells with congested vessels (Figure 6B1). In addition, a severe interstitial inflammatory reaction could be seen between muscle bundles (Figure 6B2). Furthermore, the section in the celecoxib-treated group (group III) showed no inflammation: muscles were normal; however, the epidermis was thickened and covered with excessive keratosis with underlying excessive collagenosis (Figure 6C). The section in YGME-100-treated group (group IV) showed superficial dermal mild chronic inflammation with mild edema and mild vascular congestion. Moreover, the epidermis was covered with excessive keratosis (Figure 6D). However, the sections of the YGME-200-treated group (group V) showed normal skin without inflammation or collagenosis and normal epidermis lined with thick keratin and dermis (Figure 6E).

#### 3.5.8. Effects of YGME on the Immunohistochemical Staining of COX-2 in Paw Tissues

**Figure 7:**
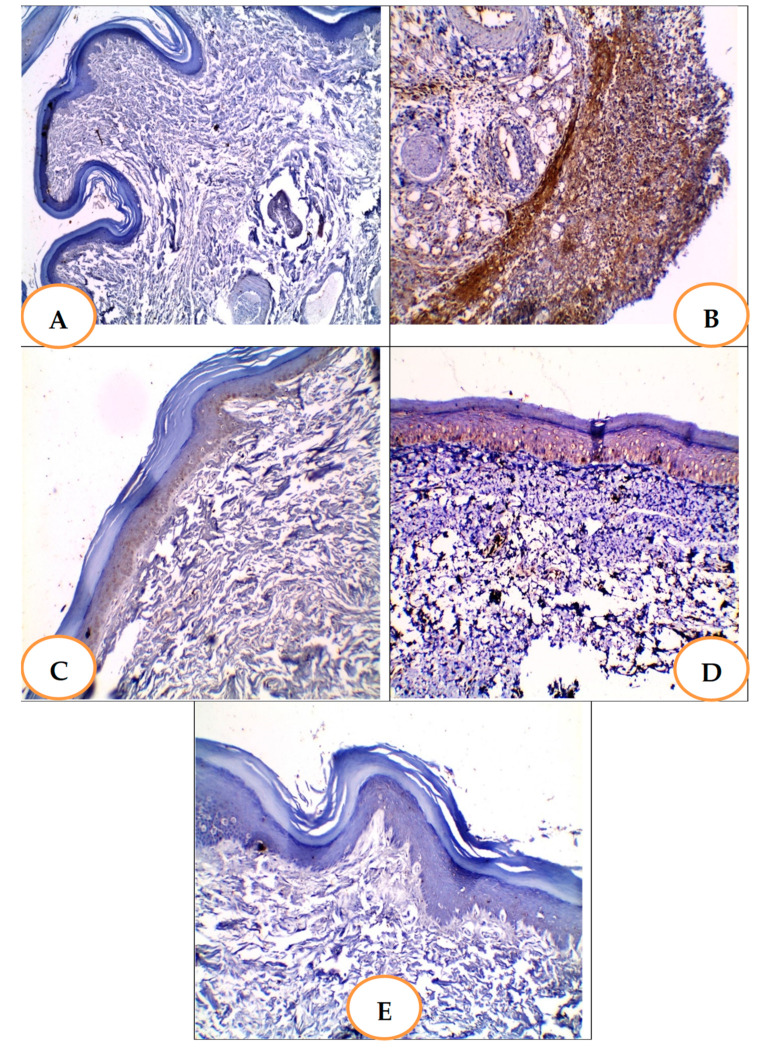
Effects of different treatments on immune histochemical staining of COX-2 in paw tissues. (**A**): Section in paw tissue of normal control group showed negative COX2 immunostaining [×100]. (**B**): Section in paw tissue of Carrageenan group [positive control group] showed positive strong COX-2 immunostaining score 3 [×100]. (**C**): Section in paw tissue of Celecoxib-treated group showed positive mild COX-2 immunostaining score 1 [×100]. (**D**): Section in paw tissue of YGME 100-treated group showed moderate, positive COX-2 immunostaining score 2 [×100]. (**E**): Section in paw tissue of YGME-200-treated group showed negative COX2 immunostaining [×100].

The section in the normal control group showed negative COX2 immunostaining **(**Figure 7A). The section in carrageenan (positive control group) showed a positive strong COX-2 immunostaining score 3 (Figure 7B). The section in celecoxib 50 group showed a positive mild COX-2 immunostaining score 1 (Figure 7C).

The section in the YGME100-treated group showed a moderate, positive COX-2 immunostaining score of 2 (Figure 7D). The section in the YGME-200-treated group showed negative COX2 immunostaining (Figure 7E).

## 4. Discussion

The inflammation process is a cause of discomfort and a key driver of pathophysiological events that eventually induce disease progression [14]. The carrageenan-induced paw edema is a useful model for evaluating the anti-inflammatory action of a high number of natural and synthetic products [42,43]. This is a strong chemical that induces the release of several inflammatory and pro-inflammatory mediators.

Acute inflammation is a double-phase process. The initial stage begins with the liberation of numerous mediators after carrageenan injection, while the second one is associated with prostaglandins’ release in 2–3 h [11].

The major goal of this research is to alleviate the inflammatory response in a model of acute inflammation using natural products from medicinal plants such as Yucca species. Several studies have investigated the anti-inflammatory, anti-arthritic and anti-oxidant effects of numerous Yucca species [44,45]. Yucca polyphenolics have free-radical scavenging activity, which could suppress the ROS and halt inflammatory responses [46]. They have been investigated to suppress iNOS expression, and thus inhibit NO production [47]. They also inhibit NFkB, which, in turn, stimulates iNOS, which produces the inflammatory agent nitric oxide [47]. This may clarify the anti-inflammatory effects of Yucca species.

This is the premier study investifating the anti-inflammatory and antioxidant activities of *Yucca gigantea* methanol extract (YGME) in carrageenan-induced hind paw edema. The activation of the cyclooxygenase-2 pathway and the production of PGE2, which are the major regulators of many cell activities and the principal mediators in the acute inflammation process, is often related to the carrageenan model. COX-2 is activated by elevated NO levels, owing to severe inflammatory responses in a variety of chronic inflammatory diseases [48]. In the current study, the carrageenan induced a strong, positive immunostaining of COX-2 and up-regulated paw PGE-2 levels. Furthermore, this increase in PGE-2 levels was attenuated by celecoxib, which is a selective COX-2 inhibitor. YGME treatment significantly reduced COX-2 staining and PGE-2 levels in paw tissues in a dose-dependent manner. LC-ESI-MS/MS of YGME revealed that it contained several flavonoids, such as apigenin and quercetin, which strongly suppressed COX-2 and PGE-2 levels, and these results are consistent with References [49,50,51].

A histopathological study of paw tissue in a group treated by YGME 200 mg/kg exhibited normal skin without inflammation or collagenosis and normal epidermis lined with thick keratin and dermis.

In addition, the data from this study indicate that carrageenan induced the production of several inflammatory cytokines, such as TNF-α, IL-1β, and IL-6, which are dose-dependently attenuated by YGME 100 and 200, and the results were equivalent to celecoxib, confirming the anti-inflammatory results of YGME. In addition, carrageenan induced a marked neutrophil infiltration, shown by a significant increase in MPO activity, which was strongly attenuated by YGME, with promising potential for the use of YGME as an anti-inflammatory compound in different diseases. YGME flavonoids strongly suppressed several inflammatory mediators, which may have crucial value in the protection and treatment of various inflammatory diseases linked to the excessive production of chemical mediators [52], and these results are consistent with previous reports [50,53,54,55,56,57]

It is revealed that YGME contain apigenin and quercetin, which were found to inhibit PGE-2 [58] and NO production. The latter primarily regulated PGE-2 synthesis by preventing the production of ROS [52,59]. It was reported that quercetin suppressed the LPS-stimulated TNF-α release in RAW cells [60]. The luteolin flavonoid’s ability to inhibit protein tyrosine phosphorylation, NFĸB-mediated gene expression, and the release proinflammatory mediators in macrophages was also investivated [60]. In addition, luteolin decreased the expression of inflammatory mediators via blocking the Akt/NFκB pathway in mice with acute lung injury caused by LPS [61].

In this study, paw NO was elevated, whereas GSH was reduced, after carrageenan injection. Treatment with YGME 100 and 200 reduced paws’ NO production and restored depleted GSH levels in a dose-dependent manner. In addition, the celecoxib-treated group showed a marked decrease in paw NO content and replenished the paw’s depleting GSH levels.

Flavonoids and saponins were reported to inhibit NO production. YGME contained several flavonoids, such as luteolin and kaempferol derivatives, which were reported to have strong inhibitory effects on NO production, and these results are consistent with the literature, which reported that they inhibit NO in lipopolysaccharide (LPS)-stimulated macrophages [9,60,61,62]. Finally, these findings could explain the anti-inflammatory and antioxidant effects of YGME on the acute inflammation model.

Free radical generation at the inflammation site causes major tissue damage, accompanied by many inflammatory disorders [14]. NO produced by inducible nitric oxide synthase (iNOS) was a principle arbitrator in the inflammation process and played a pivotal role in aggravating inflammation and NO-mediated cytotoxicity [13]. Therefore, in this study, we assessed the cytotoxic effect of YGME using SRB cell viability assay to find this extract’s ability to prevent and treat cancer in addition to reducing inflammation. YGME was topically applied to protect skin and treat different skin infections and diseases [19].

The SRB assay revealed that the YGME exerted a cytotoxic effect against all tested cell lines. The best cytotoxic effect was against human melanoma cell line (A375) and osteosarcoma cell line (MG-63). The results showed that YGME can reduce inflammation, e.g., arthritis, and has anti-osteosarcoma as well as anti-melanoma effects. The selectivity index was calculated to be 1.73, 1.41, 1.72, and 0.87 of YGME against A375, Hep-2, MG-63, and A431, respectively. Nutraceuticals include active phytochemical components as flavonoids in plants, and are used as an integrative treatment in up to 82% of patients suffering from cancer. Flavonoids are the most important group of active constituents, composed of flavones, flavonols, flavanones, flavanols, and isoflavonols [63]. LC-ESI-MS/MS of *Y. gigantea* revealed the presence of hesperetin, naringenin, quercetin-4′-*O*-glucoside, luteolin, kaempferol glycosides, caffeic acid, and methoxylated flavones. It was reported that hesperetin exerted an apoptotic effect towards A431 epidermoid skin carcinoma through cyclin and MAPK regulation. Naringenin was found to be effective against A431 cancer. Hesperidin is reported to be more cytotoxic than neohesperdin and naringin against HepG2, as well as showing anti-lung-cancer activity against the A549 cell line [64]. The apoptotic effect of luteolin and quercetin was evidenced in TRAIL-resistant cancer cells. They showed, besides kaempferol, an antiproliferative effect against VCAR3 ovarian cancer [65]. Quercetin exerted cytotoxicity against different types of cancers [66]. Polyphenols such as caffeic acid are antioxidants; they showed an anticancer effect by inhibiting DNA methylation, cyclooxygenase, and DNA topoisomerase enzymes, in addition to influencing cell signaling [65]. Benzo(a)pyrene or B(a)P is a common cancer-promoter through its bioactivation to a form that eventually binds to DNA, causing cancer initiation. Methoxylated flavones, e.g., 3′,4′-dimethoxyflavone, and 5,7-dimethoxyflavone, were evidenced to inhibit the bioactivation of B(a)P in vitro. 5,7-dimethoxyflavone could strongly bind to DNA in cell culture, and inhibit the bioactivation of B(a)P in HepG2 cell line [67,68].

Previous studies on innate immunity suggested that intestinal inflammation could be caused by bacterial infections in genetically susceptible hosts [69]. Infectious agents such as Gram-positive and Gram-negative bacteria could activate inflammatory cells and trigger inflammatory signaling pathways [70]. At present, bacterial infections are a critical threat, owing to the spread of antibiotic resistance, which has been linked to the misuse and overuse of antibiotics alongside the lack of development of new medications [71]. The emergence and dissemination of antibiotic-resistant bacteria have reduced the effectiveness of the currently used antibiotics for treatment and raised the need for the exploration of new antimicrobials from natural sources [72]. This study provided evidence for the antimicrobial potential of YGME extract, which could be utilized as an alternative to antimicrobials, as it is obvious from our results that YGME extract has a broad spectrum of antimicrobial activity. As shown in Table 2, YGME exhibited a promising antimicrobial activity against bacteria (both Gram-positive and Gram-negative). In addition, it revealed antifungal activity against *Candida albicans* as a representative for fungi. Thus, the antimicrobial activity of YGME should be investigated in more depth in future studies to elucidate its mechanism of action.

The antimicrobial effect of YGME against Gram-positive, Gram-negative, and fungi could prevent inflammation caused by an infectious pathogen. As well as its anti-inflammatory effect, it could prevent various disorders and chronic health conditions, such as cancer and cardiovascular diseases. A low-grade inflammatory state is correlated with these ailments [73]. The multiple biological effects of YGME may help transnational industries in the development of new, curative drugs from extraordinary plants such as *Y. gigantea*.

## 5. Conclusions

The findings of this study indicate that methanol extract from the leaves of *Yucca gigantea* alleviated inflammation and oxidative stress in the carrageenan-induced acute inflammation model in rats. This was shown by a significant reduction in paw COX-2 staining, PGE-2 levels, inflammatory cytokines IL-1β, TNF- α, and IL-6 levels. It also strongly suppressed NO production and restored GSH antioxidant capacity. Using LC-MS/MS to investigate the chemical composition of YGME, 29 compounds were tentatively identified from different phytochemical classes, such as saponins, flavonoids, organic and phenolic compounds. Flavonoids were reported to have anti-microbial, anti-inflammatory, antioxidants, and cytotoxic effects, including apigenin, kaempferol, luteolin, and quercetin, which could explain the beneficial effects of YGME in infectious, inflammatory, and cancer conditions. YGME exhibited cytotoxic activity against all the investigated cell lines, especially against A-375 and MG-63 cell lines. YGME also presented a potent activity against Gram-negative, Gram-positive isolates, and the fungal isolate *Candida albicans.* The antimicrobial effect of YGME could prevent inflammation caused by an infectious pathogen. In addition, its ability to reduce anti-inflammatory effects could prevent cancer and cardiovascular diseases. Therefore, Y*ucca gigantea* Lem. may have a promising future in the natural drug discovery of anti-inflammatory, antioxidants, antimicrobial, and cytotoxic drugs.

## Figures and Tables

**Table 1:** Phytochemical profiling of YGME by LC-ESI-MS/MS in negative mode.

No.	Rt (min.)	[M − H]^−^ *m*/*z*	MS^2^ *m*/*z* or Fragments	Formula	Idenification
1	1.128	115.000	115.001, 89.023, 71.012	C_4_H_4_O_4_	Maleic acid
2	1.348	449.085	449.088, 269.015, 113.019	C_21_H_22_O_11_	Okanin-4′-*O*-glucoside (Marein)
3	1.398	129.019	129.019	C_5_H_6_O_4_	Citraconic acid
4	1.411	141.018	141.020, 97.028	C_6_H_6_O_4_	Muconic acid
5	4.37	739.790	739.792, 577.415	C_39_H_61_O_13_	Spirostan-3-ol-glucoside-galactoside
6	4.77	429.171	429.171	C_27_H_42_O_4_	Hecogenin
7	4.901	593.152	593.156, 473.105, 341.111, 285,251	C_27_H_30_O_15_	Kaempferol-7-*O*-neohesperidoside
8	5.64	431.192	431.163, 392.918, 385.182, 341.164, 324.942	C_21_H_20_O_10_	Kaempferol-3-*O*-α-l-rhamnoside
9	6.22	739.790	739.792, 577.415	C_39_H_61_O1_3_	Spirostan-3-ol-diglucoside
10	6.239	447.09	447.090, 285.214, 248.960	C_27_H_30_O_16_	Luteolin-7-*O-*β-d-glucoside
11	6.276	577.156	577.160, 413.081, 311.048, 293.046	C_27_H_30_O_14_	Vitexin-2″-*O*-rhamnoside
12	6.495	609.147	609.143, 301.023	C_28_H_34_O_15_	Hesperetin-7-*O*-neohesperidoside
13	6.659	315.135	315.197, 287.198	C_16_H_12_O_7_	3′-Methoxy-4′,5,7-trihydroxyflavonol (Isorhamnetin)
14	6.817	463.085	463.089, 354.916, 326.930, 301.041, 286.936	C_21_H_20_O_12_	Quercetin-4′-*O*-glucoside
15	6.978	623.197	623.159, 577.163, 315.049	C_28_H_32_O_16_	Isorhamnetin-3-*O*-rutinoside
16	7.093	593.519	593.149, 285.030, 241.042	C_30_H_26_O_13_	Kaempferol-3-*O*-(6-p-coumaroyl)- glucopyranoside
17	7.263	593.265	593.149, 570.246, 547.242, 285.038	C_28_H_34_O_14_	Isosakuranetin-7-*O*-neohesperidoside (Poncirin)
18	8.182	285.077	285.071, 179.037, 165.016, 119.049	C_15_H_10_O_6_	4′,5,7-Trihydroxyflavonol
19	9.458	301.069	301.073, 273.072, 139.048	C_16_H_14_O_6_	Hesperetin
20	10.199	271.060	271.064, 196.049, 165.024, 151.005	C_15_H_12_O_5_	Naringenin
21	10.488	431.17	431.170, 269.040, 253.054	C_15_H_10_O_5_	Apigenin-7-*O*-β-d-glucoside
22	10.876	285.039	285.065, 256.039, 179.033, 145.030	C_15_H_10_O_6_	Luteolin
23	10.992	179.033	179.037, 135.042	C_9_H_8_O_4_	Caffeic acid
24	11.313	283.060	283.064, 211.070, 189.021, 177.014	C_15_H_12_O_6_	Acacetin
25	11.531	315.092	315.086, 297.075, 193.009, 179.033, 152.010	C_16_H_12_O_7_	3,3′,4′,5-tetrahydroxy-7-methoxyflavone
26	12.594	299.093	299.090, 193.049, 149.058	C_16_H_12_O_6_	3,5,7-Trihydroxy-4′-methoxyflavone
27	13.760	461.260	461.262	C_27_H_44_O_3_	25 R or S-Spirostanol-3-ol
28	14.034	223.171	223.163, 113.991	C_11_H_12_O_5_	3-(4-Hydroxy-3,5-dimethoxyphenyl)-2-propenoic acid
29	19.75	277.197	277.218, 276.367, 259.204, 233.224, 205.203	C_18_H_30_O_2_	gamma-Linolenic acid

**Table 2:** The antimicrobial potential of YGME against different microbial isolates.

Pathogenic Bacterial Isolate	Inhibition Zone Diameter (mm)		MIC Values (µg/mL)
	YGME	Chlorhexidine	
	Gram-negative bacteria		
*Klebsiella pneumoniae*	12.5 ± 0.41	27.5 ± 1.35	106.67 ± 30.1
*Escherichia coli*	13.3 ± 0.65	28.5 ± 1.50	85.3 ± 30.0
*Pseudomonas aeruginosa*	10.2 ± 0.69	23.4 ± 0.77	42.6 ± 15.0
*Proteus mirabilis*	14.8 ± 0.33	26.4 ± 0.89	74.67 ± 39.9
*Salmonella typhimurium*	10.5 ± 0.42	23.3 ± 0.85	53.3 ± 15.08
*Pseudomonas aeruginosa*	10.2 ± 0.69	23.4 ± 0.77	42.6 ± 15.0
	Gram-positive bacteria		
*Staphylococcus aureus*	14.46 ± 1.14	24.9 ± 1.40	85.3 ± 30.0
*Staphylococcus epidermidis*	16.5 ±0.75	26.6 ± 0.98	106.67 ± 30.1
	Fungi		
*Candida albicans*	12 ± 1.3	17.8 ± 2.1	21.3 ± 7.5

**Table 3:** The average change in the edema volume at different time intervals over 4 h.

Time (h)	The Average Change in Edema Volume (mm) *			
	Group II	Group III	Group IV	Group V
1	0.36 ± 0.05	0.3508 ± 0.031	0.32 ± 0.08	0.3703 ± 0.012
2	0.62 ± 0.12	0.208 ± 0.061	0.24 ± 0.031	0.2307 ± 0.02
3	0.81 ± 0.04	0.1108 ± 0.011	0.18 ± 0.04	0.1303 ± 0.03
4	1.20 ± 0.11	0.01 ± 0.001	0.12 ± 0.013	0.01 ± 0.001

* Data are represented as mean *±* SD at significant level of *p <* 0.05.

**Table 4:** Effects of YGME on the average paw edema weight, NO, GSH, and MPO activity in carrageenan-induced acute inflammation in rats.

	Average Paw Weight (g)	Paw NO Content (nmol/g Tissue)	Paw GSH Content (µmol/g Tissue)	Paw MPO Activity (µM/min/g Tissue)
Group I	0.03 ± 0.001	10.6 ± 0.89	13.94 ± 1.1	2.73 ± 0.39
Group II	0.37 ± 0.01 ^a^	20.2 ± 1.30 ^a^	9.74 ± 0.81 ^a^	13.36 ± 0.77 ^a^
Group III	0.15 ± 0.012 ^b^	10.8 ± 0.83 ^b^	15 ± 1.1 ^b^	4.48 ± 0.94 ^b^
Group IV	0.17 ± 0.014 ^b^	13.6 ± 1.1 ^b^	16.8 ± 1.3 ^b^	5.97 ± 0.0.39 ^b^
Group V	0.07 ± 0.002 ^bc^	10.5 ± 0. 5 ^bc^	23.2 ± 1.48 ^bc^	6.21 ± 0.16 ^bc^

Data are expressed as mean ± SD. Significant difference vs. ^a^ respective control saline, ^b^ respective carrageenan group, ^c^ respective YGME 100 group, each at *p* ˂ 0.05.

## Data Availability

The data are available upon request.

## Supplementary Materials

The following supporting information can be downloaded. Figure S1: TIC and BPC of LC-ESI-MS/MS analysis of *Yucca gigantea* methanol extract in negative ionization mode, Figure S2: The MS/MS spectrum of the major identified compounds, Table S1: ^1^H-NMR (DMSO-*d*_6_, 400 MHz) and ^13^C NMR (DMSO-*d*_6_, 100 MHz) for the isolated pure compounds.

## Sample Availability

Not available.
