# Supplementary material for: Elucidation of the Metabolite Profile of Yucca gigantea and Assessment of Its Cytotoxic, Antimicrobial, and Anti-Inflammatory Activities
Source: Molecules. 2022 Feb 16;27(4):1329. doi: 10.3390/molecules27041329 (PMC8878216; doi:10.3390/molecules27041329)
Supplement: Supplementary file 1 [file molecules-27-01329-s001.zip › molecules-1593259-supplementary.pdf]

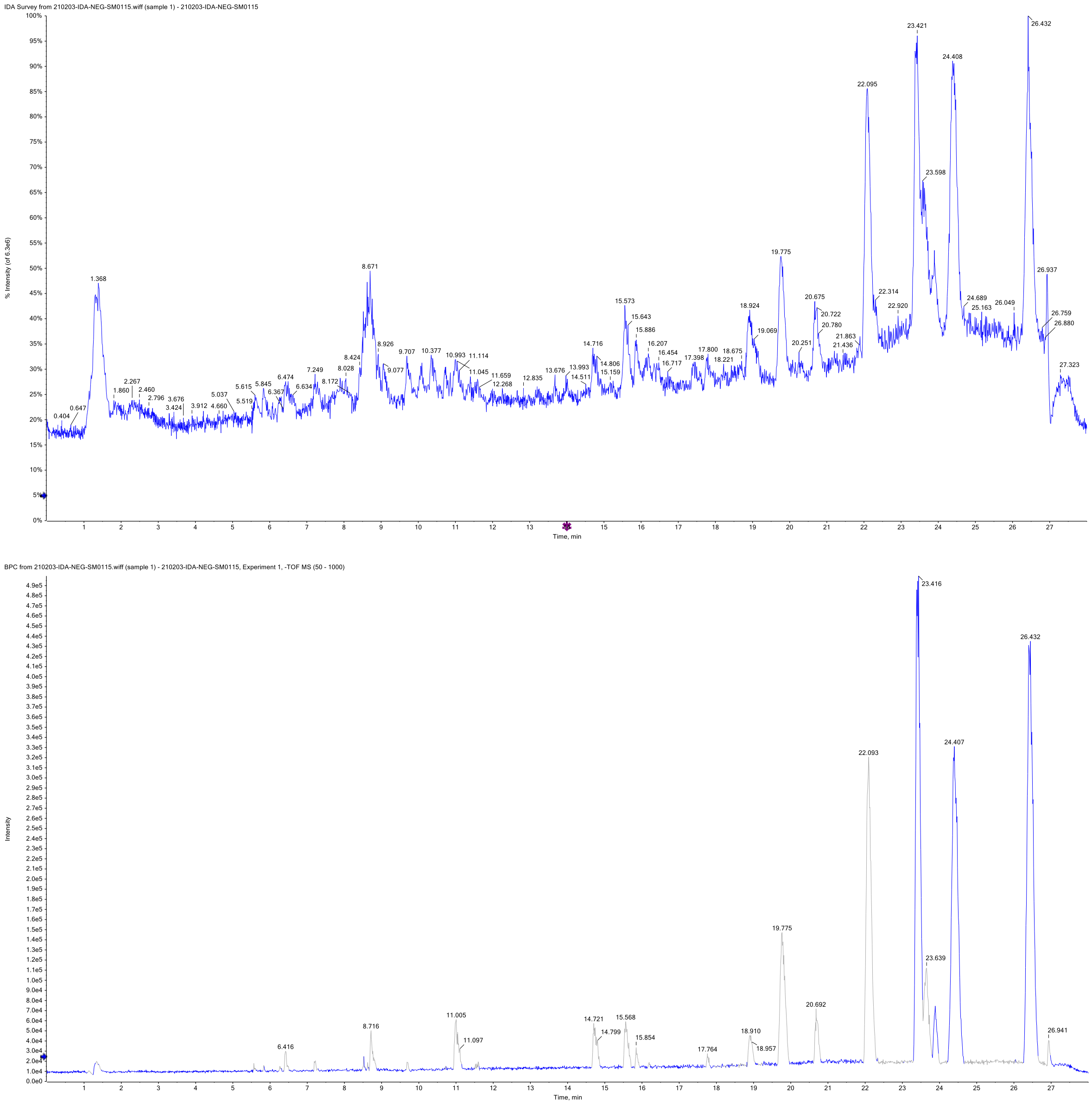

Figure S1: TIC and BPC of LC-ESI-MS/MS analysis of *Yucca gigantea* methanol extract in negative ionization mode

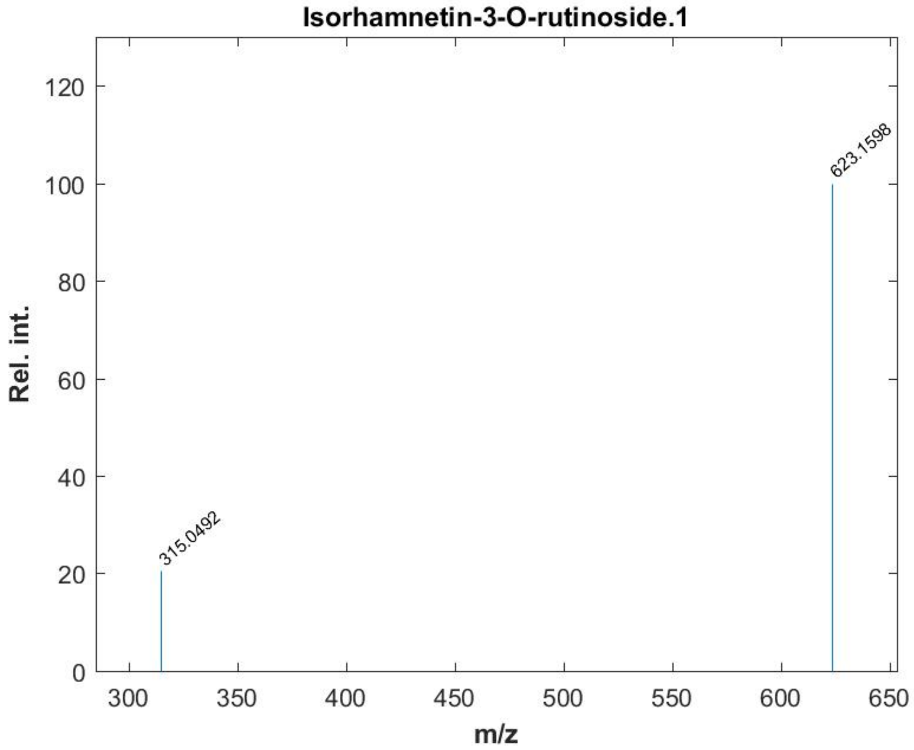
Isorhamnetin-3-O-rutinoside.1
Rel. int.
m/z
315.0492
623.1598
0
20
40
60
80
100
120
300
350
400
450
500
550
600
650

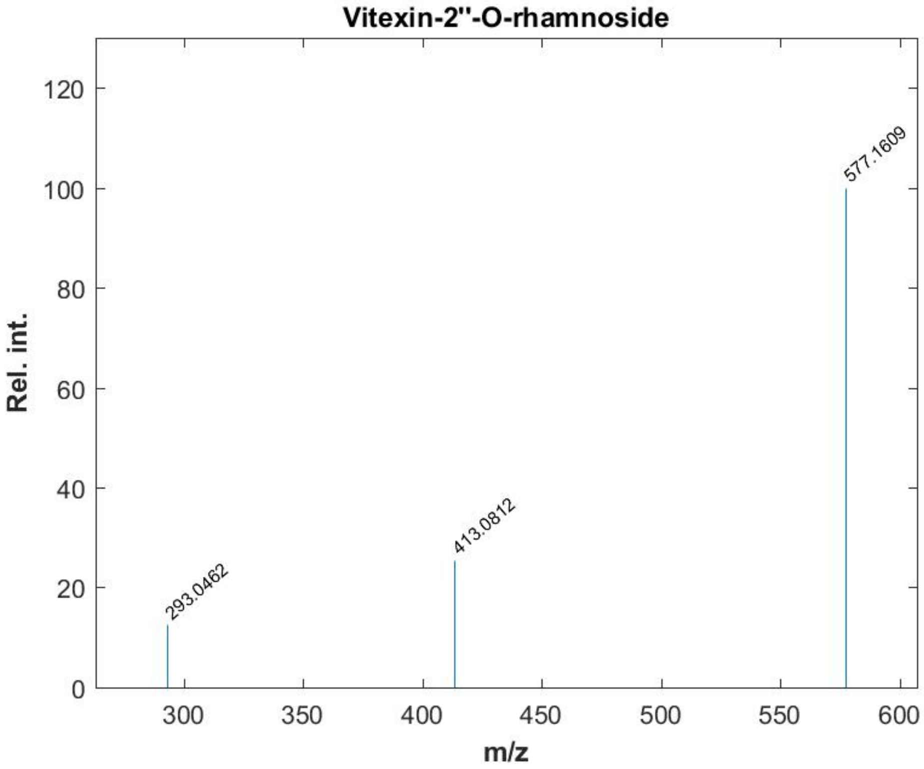
Vitexin-2''-O-rhamnoside
Rel. int.
m/z
293.0462
413.0812
577.1609
0
20
40
60
80
100
120
300
350
400
450
500
550
600

Luteolin

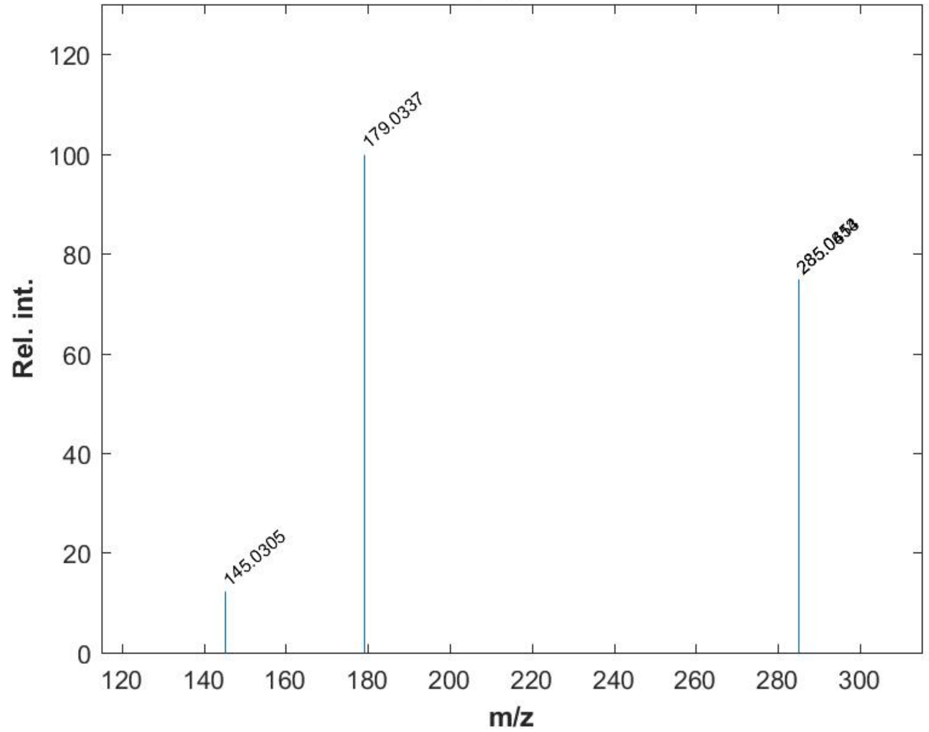

4',5,7-TRIHYDROXYFLAVONOL.1

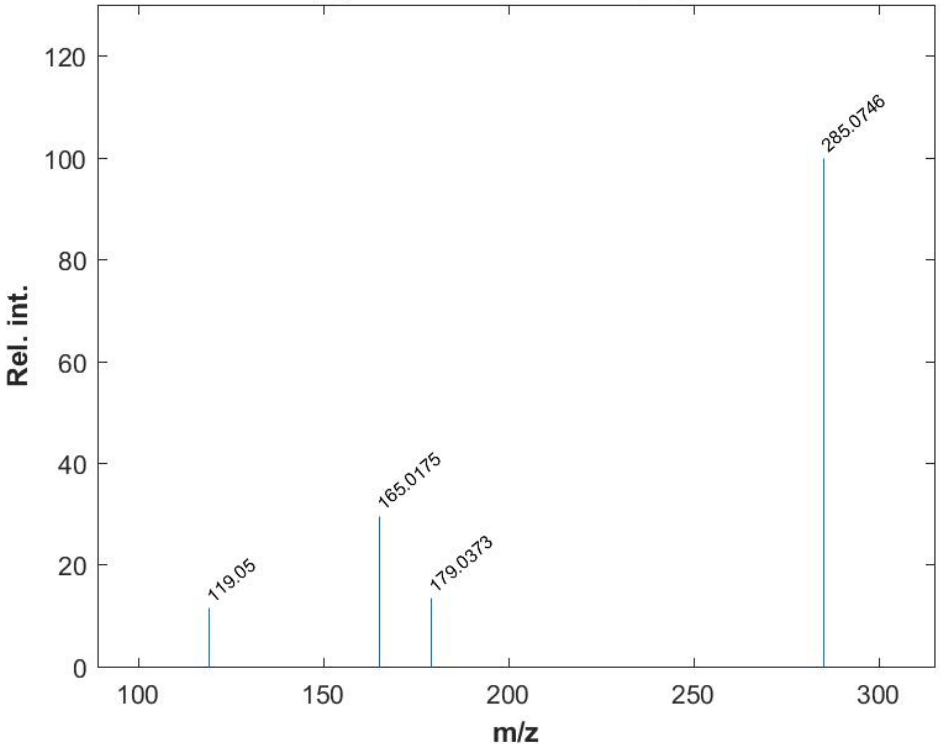

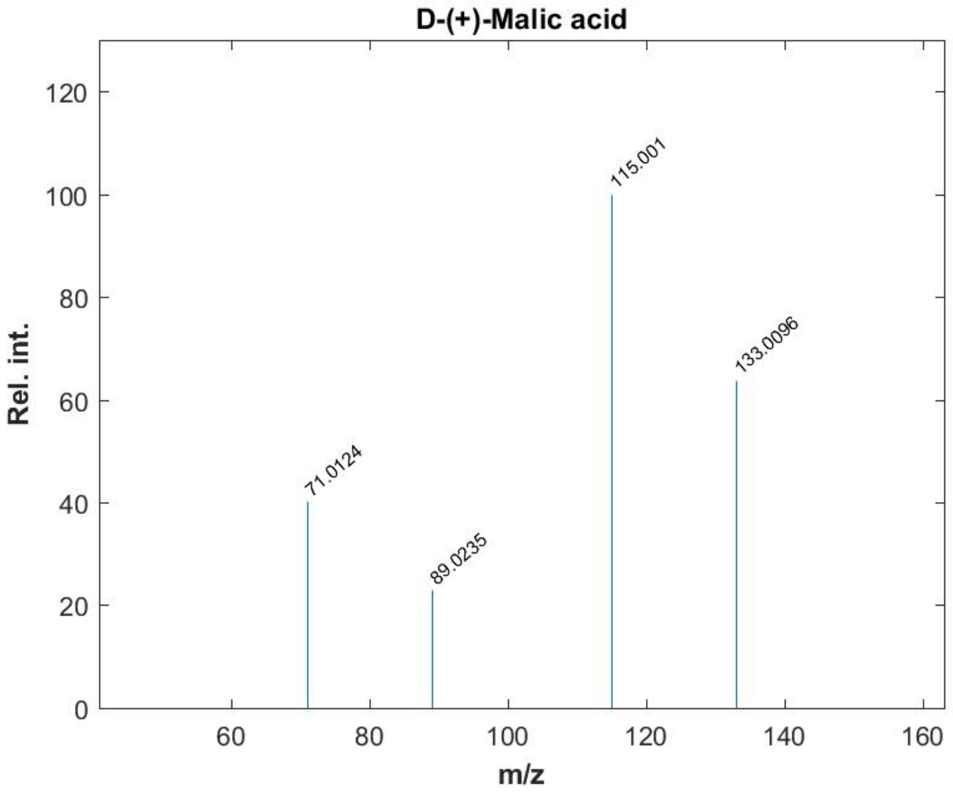

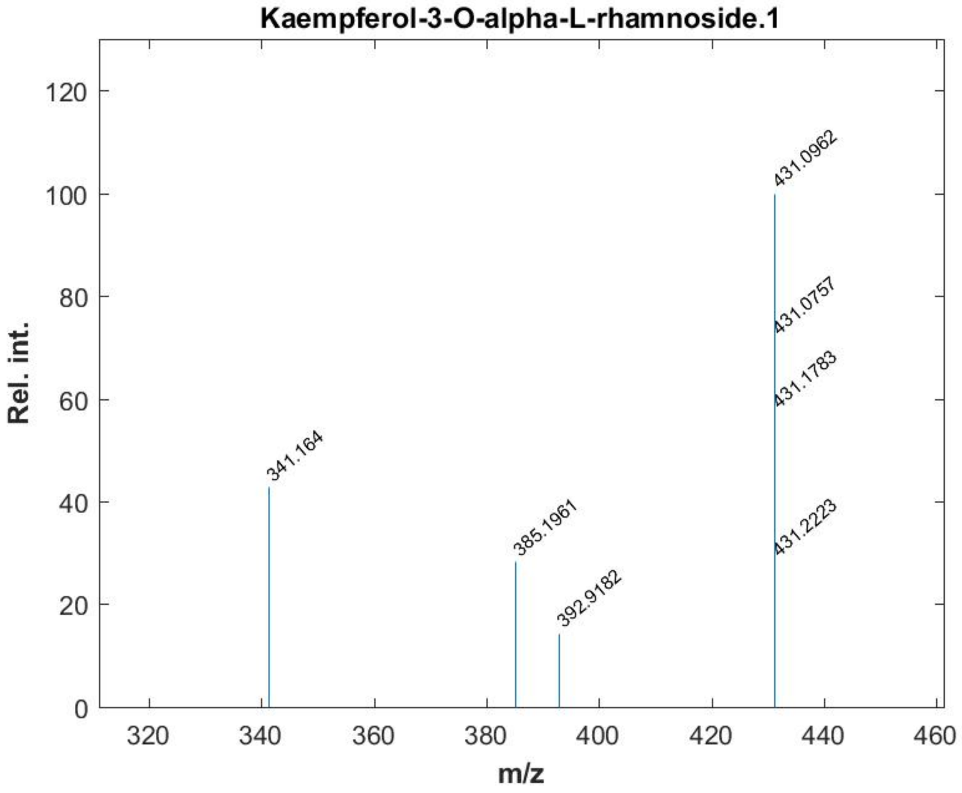

Figure S2: The MS/MS spectrum of the major identified compounds.

Table S1. ^{1}H NMR (DMSO-d_6, 400 MHz) and ^{13}C NMR (DMSO-d_6, 100 MHz) for the isolated pure compounds.

	**Compound I** **Luteolin- 7-*O*- *β*-D-glucoside**		**Compound II** **Apigenin- 7-*O*- *β*-D-glucoside**		**Compound III** **Kaempferol-3-*O*- α-L-rhamnoside**	
	**δH**	**δC**	**δH**	**δC**	**δH**	**δC**
**2**		164.6		163.0		157.7
**3**	6.77 (s)	103.3	6.83 (s)	103.2		133.5
**4**		181.9		182.1		178.2
**5**		161.2		161.5		161.7
**6**	6.44 (d *J* = 2.4)	99.6	6.45 (d, *J* = 2.0)	99.6	6.21 (d, *J* = 1.6)	99.4
**7**		163.0		164.3		164.1
**8**	6.78 (d *J* = 2.4)	94.8	6.93 (d, *J* = 2.0)	94.9	6.43 (d, *J* = 1.6)	94.2
**9**		157.0		161.5		154.9
**10**		105.4		105.4		104.6
**1'**		121.5		121.1		120.9
**2'**	7.45 (d, *J* =2.4)	113.7	7.95 (d, *J* =8.0)	128.7	7.77 (dd, *J* =8.0, 1.6)	131.0
**3'**		145.9	6.94 (d, *J* =8.0)	116.1	6.92 (dd, *J* =8.0, 1.6)	115.9
**4'**		150.0		161.2		160.4
**5'**	6.91 (d, *J* = 8.0)	116.1	6.94 (d, *J* =8.0)	116.1	6.92 (dd, *J* =8.0, 1.6)	115.9
**6'**	7.42 (dd, *J* = 8.0, 2.4)	119.3	7.95 (d, *J* =8.0)	128.7	7.77 (dd, *J* =8.0, 1.6)	131.4
**1''**	4.69 (m)	99.9	5.06 (d, *J* =7.2)	99.9	5.29 (s)	102.2
**2''**	3.71 (m)	73.2	3.72 (m)	73.2	3.98 (m)	70.6
**3''**	4.61 (m)	77.2	4.60 (m)	76.5	3.48 (m)	70.8
**4''**	5.05 (m)	69.6	5.06 (m)	69.6	3.15 (m)	71.5
**5''**	5.11 (m)	76.5	5.12 (m)	77.3	3.08 (m)	71.1
**6''**	5.39 (m)	60.7	5.38 (m)	60.7	0.79 (d, *J* =5.6)	17.9
